# Development of MicroRNAs as Potential Therapeutics against Cancer

**DOI:** 10.1155/2020/8029721

**Published:** 2020-07-15

**Authors:** Noraini Abd-Aziz, Nur Izyani Kamaruzman, Chit Laa Poh

**Affiliations:** ^1^Centre for Virus and Vaccine Research (CVVR), Sunway University, 47500 Subang Jaya, Selangor, Malaysia; ^2^Department of Biomedical Science, Faculty of Medicine, University of Malaya, 50603 Kuala Lumpur, Malaysia

## Abstract

MicroRNAs (miRNAs) are small noncoding RNAs that function at the posttranscriptional level in the cellular regulation process. miRNA expression exerts vital effects on cell growth such as cell proliferation and survival. In cancers, miRNAs have been shown to initiate carcinogenesis, where overexpression of oncogenic miRNAs (oncomiRs) or reduced expression of tumor suppressor miRNAs has been reported. In this review, we discuss the involvement of miRNAs in tumorigenesis, the role of synthetic miRNAs as either mimics or antagomirs to overcome cancer growth, miRNA delivery, and approaches to enhance their therapeutic potentials.

## 1. Introduction

Cancer is the second cause of death next to ischaemic heart disease and stroke worldwide. GLOBOCAN 2018 has estimated over 18.1 million new cancer cases and 9.6 million deaths in 2018 worldwide [1]. Abnormalities in cell growth that involve dysregulation of gene expression are able to initiate carcinogenesis. Over time, cancer cells can spread or metastasize to other parts of the body and further complicate treatments against the disease. Current treatments for cancers such as surgery, chemotherapy, and radiotherapy are not completely successful. Hence, there is a need to develop other strategies to complement conventional therapies. Numerous studies have confirmed that overexpression of microRNAs (miRNAs) has the potential to promote cancer development [2]. Intriguingly, some miRNAs have been identified to exert anticancer effects. Thus, defined interrogations of the oncogenic miRNAs (oncomiRs) that can lead to irregularities of gene expression or enhancement of tumor suppressor miRNAs might become potential therapeutic approaches.

The discovery of lin-4 miRNA in *Caenorhabditis elegans* (*C. elegans*) has led to the identification of other miRNAs in plants, animals, and humans [3]. The central dogma of molecular biology involves the transcription of deoxyribonucleic acid (DNA) into messenger ribonucleic acid (mRNA) and the translation of the mRNA into proteins. miRNAs are short sequences of noncoding RNAs (19–24 nucleotides) functioning at the posttranscriptional stage where they can regulate the protein translation process. miRNAs have the ability to bind to the complementary sequence of the mRNA at the 3′ untranslated region (UTR), and the binding of the miRNA to mRNA will halt the progression of protein translation [4]. However, the base-pairing binding does not have to be perfect binding for all the 20 nucleotides of the miRNA. Thus, a single miRNA can regulate a large number of gene expressions, and the translation of multiple mRNAs can be governed by a single miRNA. miRNAs have been reported to regulate more than 50% of human genes which are situated in cancer-associated genomic regions that form central nodal points in cancer development pathways [5, 6]. This suggests that miRNAs might play a crucial role in the pathogenesis of human cancers.

## 2. miRNAs Biogenesis

**Figure 1:**
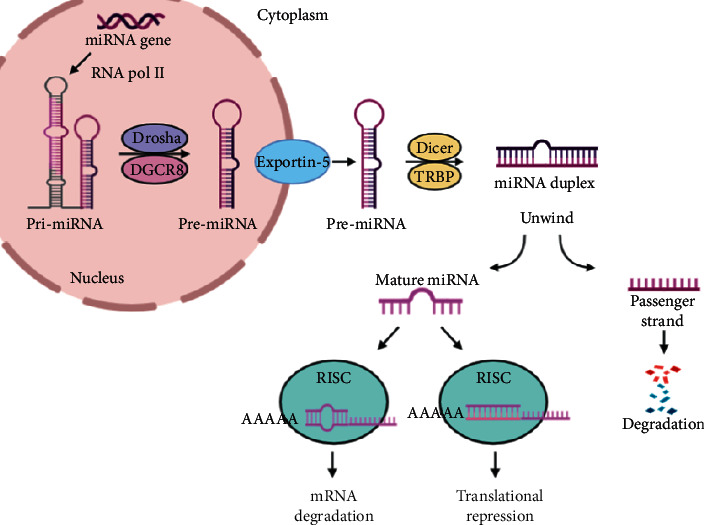
MicroRNA biogenesis pathway. miRNA genes are transcribed to produce primary miRNA transcript (pri-miRNA) by RNA polymerase II and cleaved into precursor miRNA transcript (pre-miRNA) by the microprocessor complex Drosha-DGCR8 in the nucleus. The pre-miRNA is then exported into the cytoplasm by Exportin-5 where it is processed into miRNA duplex by Dicer and its interacting partner, TRBP. The miRNA duplex is then unwound into two single-stranded miRNAs. The functional strand of mature miRNA is uploaded onto the RNA-induced silencing complex (RISC) and negatively regulates gene expression resulting in either target mRNA degradation or translation repression, while the passenger strand is degraded.

miRNAs are noncoding sequences in the DNA that are not translated into proteins. However, they have been found to function in regulating gene expression. miRNA biogenesis initially begins with transcription of the noncoding region into a large primary transcript (pri-miRNA) by the RNA polymerase II. Next, the RNase III enzyme, Drosha, will interact with the RNA specialized binding protein (DGCR8) to form the microprocessor complex, and the tails of the pri-miRNA will be removed to produce precursor miRNA (pre-miRNA). Pre-miRNA will travel across the nuclear membrane with the assistance of the Exportin-5 protein. In the cytoplasm, the RNase III Dicer will form a complex with the transactivation response element RNA-binding protein (TRBP) and cleaves the stem-loop on the pre-miRNAs, resulting in a miRNA duplex with 18–25 nucleotides in length. The duplex strand will be unwound, leaving the mature strand inside the RNA-induced silencing complex (RISC), while the passenger strand of the miRNA will be degraded [7]. RISC will travel to find the target sequence on the mRNA [8]. The region on the miRNA that can bind with perfect pairing is termed the “seed sequence,” which is mostly located at nucleotide positions 2–7 from the 5′ end of the miRNA. The perfect pairing of the miRNA with the target mRNA promotes mRNA degradation, while imperfect base pairing will lead to protein translational repression (Figure 1) [9]. Mutations in genes encoding the four key enzymes such as Drosha, Exportin-5, Dicer, and Argonaute 2 involved in miRNA biogenesis were commonly found in cancers [10].

## 3. miRNAs in Cancer

Cancer is the most significant pathology in the world of miRNA-mRNA interplay, where many of the miRNA target sites are clustered in cancer-associated genomic regions. To date, about 28,465 miRNAs have been discovered [11]. Hence, miRNA is considered as the master regulator in the cellular network. Dysregulation in expression of miRNAs has been reported to associate with the stage, progression, and metastasis of cancers [12]. miRNAs can be classified as oncomiRs or tumor suppressor miRNAs. OncomiRs are constitutively overexpressed and repressed the translation of tumor suppressor genes, leading to the promotion of tumor cell growth. Thus, high expression of an oncomiR significantly increases oncogenic properties such as cell proliferation, migration, and invasion. On the other hand, tumor suppressor miRNAs commonly suppressed the translation of mRNAs which encode for oncogenes, thereby inhibiting tumorigenesis and subsequent development of cancers. miRNAs with oncomiR or tumor suppressor functions are presented in Table 1.

The upregulated oncomiRs such as miR-21, miR-17-92 cluster, and miR-155 could increase tumorigenesis. miR-21 is one of the most commonly overexpressed oncomiRs in different types of solid tumors such as breast, lung, colon, glioblastoma, pancreatic, ovarian, prostate, and gastric cancers, as well as lymphomas [13]. miR-21 is an example of miRNAs that targets multiple oncogenic signaling cascades as well as causing global dysregulations of gene expression in cancers. Overexpression of miR-21 has been demonstrated to target an array of tumor suppressor genes such as programmed cell death 4 (PDCD4), phosphate and tensin homolog (*PTEN*), *RECK*, and tropomyosin 1 (TPM1) [13–16]. This process promoted cell proliferation, metastasis, invasion, and chemoresistant phenotypes.

Another oncomiR, miR-17-92 cluster, was also transcriptionally upregulated in several types of cancers including lymphomas, lung, colon, and gastric cancers [18]. The miR-17-92 cluster comprises miR-12, miR-18a, miR-19a, miR-20a, miR-19b, and miR-92a, and it was reported to facilitate tumor proliferation and induce angiogenesis through the activation of c-Myc commonly activated in cancers [18, 19].

miR-155 is one of the eminent tumor-promoting miRNAs that were found to be overexpressed in lymphoma, lung, breast, and ovarian cancers. High levels of miR-155 were associated with poor prognosis [20, 21]. Previous studies showed that overexpression of miR-155 directly downregulated SHIP1 and C/EBP*β* genes, which consequently blocked B-cell differentiation and enhanced cell survival [38, 39]. Besides, miR-155 also downregulated the expression of tumor suppressor protein, von Hippel–Lindau (VHL), leading to the induction of angiogenesis and promoted cancer cell survival [22].

Another miRNA with a distinct role in cancer pathology is miR-10b, which is involved in the late stage of malignancy, where it promotes invasion and metastasis of cancer cells. Overexpression of this miRNA has been found in glioblastoma, breast, and esophageal cancers [23–25]. This miRNA positively regulated cell migration and invasion by reducing the expression of the tumor suppressor gene, Kruppel-like factor 4 (KLF4) in human esophageal cells [25]. On the other hand, miR-10b also downregulated a member of the homeobox DNA-binding domain transcription factors known as HOXD10 in breast and ovarian cancers, resulting in a prometastatic phenotype [40, 41]. miR-221 and miR-222 are highly homologous, and their upregulation was demonstrated in several types of human tumors. The overexpression of miR-221/222 was shown to enhance cell growth, migration, and invasion in breast, lung, and liver cancers by downregulating PTEN [42].

In contrast, miRNAs such as the let-7 and miR-34 family are known to inhibit the translation of mRNAs encoding oncoproteins that regulated apoptosis or cell differentiation [43, 44]. Hence, they could prevent tumor development, and they are known as tumor suppressor miRNAs. The expression of let-7 was reduced in colon, breast, and lung cancers and was associated with poor survival [45–47]. The upregulation of let-7 has been demonstrated to suppress the growth of lung cancers *in vitro* [48]. Previous studies demonstrated that the downregulation of let-7 increased prooncogene RAS protein expression in lung tumors [28, 29].

Three members of the miR-34 family, miR-34a, miR-34b, and miR-34c were downregulated in lung, breast, colon, and several other cancers [30–33]. They were transcriptionally regulated by the tumor suppressor p53 [30]. miR-34a played an important role in p53-mediated apoptosis upon DNA damage by direct targeting of the antiapoptotic proteins Bcl-2 and SIRT1 [31]. In addition, Liu et al. showed that miR-34a could inhibit prostate cancer stem cells and metastasis by directly repressing CD44 [32]. The depletion of miR-34a expression was correlated to metastasis and recurrence in cancer, whereas restoration of miR-34a expression was correlated to apoptosis and improved the efficacy of chemotherapy and radiation.

Another group of tumor suppressor miRNAs, miR-15a and miR-16, was first reported to be aberrantly expressed in cancers in 2002, and loss of these miRNAs was shown to be associated with poor prognosis in chronic lymphocytic leukemia and colon cancer patients [34, 35]. The miRNA-29 family (miR-29a, miR-29b, and miR-29c) was demonstrated to be aberrantly expressed in multiple cancers. Evidence has shown that downregulation of the miR-29 family is correlated with tumorigenesis as well as cancer progression [36]. Fabbri et al. [37] reported that miR-29 could function as a tumor suppressor by interfering with the methylation of tumor suppressor genes, where this miRNA targeted the enzymes involved in DNA methylation (DNA methyltransferases 3A and 3B) that were upregulated in lung cancer. miR-29 was able to activate re-expression of methylation-silenced tumor suppressor genes including the fragile histidine triad protein (FHIT) and WW domain-containing oxidoreductase (WWOX) [37].

Numerous *in vitro* as well as *in vivo* studies have reported that repression of miRNA expression could promote tumorigenesis. The activity of the oncogenic or tumor suppressor miRNAs was not restricted by the type of tumor or its origin as the tumor cells could proliferate and metastasize to distant organs [49, 50]. Although the examples given comprise a small subset of miRNAs involved in the development of cancers, they underline the concept that targeting abnormally expressed miRNAs could have a promising impact on the development of future cancer therapies.

## 4. Mechanism of miRNA Deregulation in Cancer

Tumor cells have been known to have deregulated expression of miRNAs [4, 51]. Increased or reduced levels of miRNAs in tumors generally resulted in genetic abnormalities, alterations in epigenetic and transcriptional regulation, or defects in their miRNA biogenesis pathway [51]. For example, the loss of the miR-15a and miR-16-1 cluster at chromosome 13q14 is one of the reasons for miRNA deregulation in cancer. Tumorigenesis is generally correlated with chromosomal aberrations such as amplification, deletion, or translocation of specific genomic regions surrounding miRNA genes. Genome-wide analysis reported the abundance of miRNA genes that were located in cancer-associated genomic regions or fragile sites, in minimal regions of heterozygosity loss, minimal regions of amplification, or general breakpoint regions [52].

The expression of miRNAs is known to be closely regulated by various transcription factors. Transcription factors might activate miRNAs by inducing the transcription of pre-miRNAs. This process is well reported in certain cases where tissue-specific miRNA is activated by transcription factors during differentiation. The relationships between miRNA and transcription factors have been identified in cancers, and altered expression of the key transcription factors such as c-Myc, p53, and E2F was discovered to lead to deregulated expression of miRNA that could promote tumor development [53–57].

In addition, epigenetic alterations could also affect miRNA expression. The alteration would involve genomic DNA hypomethylation, abnormal DNA hypermethylation of tumor suppressor genes, and disruption of the histone modification patterns [58]. In most cancers, hypermethylation of CpG islands in promoter regions could lead to heritable transcriptional silencing of tumor suppressor genes. Fazi et al. demonstrated that miR-223 expression was epigenetically silenced by AML1/ETO, a most prevalent acute myeloid leukemia-associated fusion protein through CpG methylation [59]. Besides, Saito et al. reported that 17 of 313 human miRNAs were upregulated in T24 bladder cancer cells after concurrent treatment with DNA methylation and histone acetylation inhibitors [60]. Among all miRNAs, miR-127 embedded in a CpG island with reduced expression in cancer cells had significantly increased expression after the treatments, followed by the downregulation of protooncogene BCL6. These data revealed that DNA demethylation and histone deacetylase inhibition were able to activate the expression of miRNAs that might act as tumor suppressors. In another study, Lujambio et al. showed that CpG methylation correlated with the silencing of miR-148a and miR-34b/c due to hypermethylation in tumors [61]. The restoration of these miRNAs in the tumor was correlated with the hindrance of motility, tumor growth, and metastasis *in vivo*. Thus, these results demonstrated the function of epigenetic regulation in miRNA expression during tumorigenesis.

miRNA biogenesis is controlled by a few enzymes and regulatory proteins such as Drosha, Dicer, DGCR8, Argonaute proteins, and Exportin-5 that allow correct miRNA maturation from primary miRNA precursors. Thus, mutation or abnormal expression of any part of the miRNA biogenesis system might lead to aberrant expression of miRNAs which were associated with poor prognosis and tumor progression [62, 63].

## 5. Therapy Targeting miRNAs in Human Cancer

Since miRNAs are involved in tumor proliferation, invasion, and metastasis, miRNA-based gene therapy is becoming a new strategy for cancer treatment. There are two therapeutic strategies intended to re-establish the physiological miRNA expression in tumor cells, either by inhibiting the miRNA activity when the oncomiR is overexpressed or by restoring the miRNA activity when the tumor suppressor miRNA is repressed.

### 5.1. miRNA Inhibition Therapy

miRNA inhibition therapy is applied to repress the function of oncomiRs that are significantly upregulated in tumor cells and help to restore the normal expression and function of tumor suppressor genes. OncomiRs can be inhibited by applying antisense anti-miR oligonucleotides (AMO), locked nucleic acid (LNA), miRNA antagomirs, and miRNA sponges [64]. The fundamentals of these approaches consist of miRNA inhibitors which are essentially complementary to the single-stranded oligonucleotide and are able to isolate the endogenous miRNA in an unrecognizable structure, leading to inactivation and eliminating the mature miRNAs from the RISC.

AMOs are single-stranded, chemically modified antisense oligonucleotides that are about 17–22 nucleotides and were designed to be complementary to a miRNA of interest [65]. This antisense oligonucleotide was designed to bind to the complementary mature miRNA and inhibited the interaction of that miRNA with its specific mRNA targets, thereby allowing normal translation. LNA is an example of a modified AMO [66]. LNA-modified antisense oligonucleotides were able to display higher thermal stability and affinity with their miRNA target molecules, had higher aqueous solubility, and increased metabolic stability for *in vivo* delivery [67]. An *in vivo* study by Griveau et al. demonstrated that LNA-modified antisense oligonucleotides were capable of silencing overexpressed miR-21 in glioblastomas, leading to a significant reduction in cell viability as well as the elevated intracellular level of caspase [68].

Antagomirs are chemically modified single-stranded 23 nucleotide RNA molecules complementary to the targeted miRNAs to enhance the stability of the RNA and prevent it from degradation [69, 70]. Ma et al. reported that the silencing of miR-10b using antagomir inhibited metastasis in a mouse mammary tumor model. They also reported that silencing of this miRNA with antagomirs significantly decreased miR-10b levels and induced the levels of a functionally vital miR-10b target, HOXD10 [41].

Besides LNA treatment, miRNA sponges have also been used to inhibit oncomiRs. miRNA sponges are a class of RNAs that contain multiple artificial miRNA binding sites that compete with endogenous miRNA targets for miRNA binding [71]. In breast cancer cells, miR-9 was overexpressed and hindered the expression of CDH1, a tumor suppressor gene. miRNA sponges containing four miR-9 binding sites were able to efficiently block the function of miR-9 and restored endogenous expression of CDH1, which consequently inhibited metastasis [72]. Recently, a newly designed artificial miRNA sponge has been produced. Driven by natural circular RNA (circRNA) documented as endogenous miRNA sponges, a functional artificial circRNA sponge using a simple enzymatic ligation method was synthesized. This artificial circRNA molecule was designed as an exogenous miRNA inhibitor that efficiently bound and inhibited mature RNA, thus displaying therapeutic potential [73]. One circRNA may regulate one or more miRNAs via different miRNA binding sites in a circular sequence. Liu et al. designed circRNA sponges for miR-21 and miR-221, which were carried in a circular sponge-producing vector, and it was reported to be more effective in inhibiting miRNA targets compared to linear sponges in malignant melanoma cell lines. Transfection of the circRNA targeting miRNA-21 was shown to inhibit proliferation of gastric cancer cells by inducing apoptosis and deregulating global protein expression [74].

### 5.2. miRNA Restoration Therapy

miRNA restoration therapy is used to induce apoptosis or inhibit the proliferation of tumor cells by restoring exogenous tumor suppressor miRNAs that are downregulated in tumor cells. miRNA restoration therapy can be implemented by using synthetic miRNA mimics or employing viral vectors expressing miRNAs. miRNA mimics are able to restore the normal function of endogenous miRNAs by replacing the loss of miRNAs. The chemically modified RNA duplexes could be loaded into RISC to provide the downstream inhibition of the target mRNAs [64]. Various studies have demonstrated the efficiency of miRNA restoration therapy *in vitro* and *in vivo*. For instance, the addition of miRNA mimic miR-15 in prostate cancer cell lines caused significant apoptosis and inhibited proliferation [75]. The *in vivo* study in K-ras mutant mouse by intranasal administration of let-7 efficiently restrained the growth of the tumors by blocking cell proliferation and cell cycle pathways [76, 77].

Another strategy of miRNA restoration therapy is to use miRNA expression vectors such as adenoviral, lentiviral, and retroviral vectors to increase the expression of the miRNAs which lead to tumor suppression. Kota et al. reported the loss of expression of miR-26 in human liver cancers, even though it was expressed at high levels in normal tissues [78]. Ectopic expression of this miRNA in liver cancer cell lines was demonstrated to induce cell cycle arrest. An intravenous injection of recombinant adenovirus carrying the miR-26 resulted in inhibition of tumorigenicity by enhancing tumor apoptosis and repressing cell growth without any sign of toxicity.

## 6. Challenges in Developing miRNA-Based Therapeutics

The use of miRNA for cancer therapy has gained great attention in recent years. Despite having some advances in using miRNAs for inhibition and restoration therapies in preclinical studies, challenging hurdles remain for their successful delivery. The main hurdle of miRNA therapy in cancers is to deliver miRNA antagonists or miRNA mimics to the target tumor tissues with effective penetration into the tumor mass. The compression of abnormal tumor vessels as well as the leaky structures contributed to poor blood perfusion that could diminish the efficacy of delivery of the naked miRNA [79].

Another challenge of miRNA delivery is to sustain the integrity and stability of miRNAs in circulation. The unmodified or naked miRNAs are rapidly degraded within seconds by serum nucleases such as RNase A-type and are cleared in the blood circulation. Besides, naked miRNAs are rapidly cleared by renal excretion, which results in a short half-life in the systemic circulation [80]. In addition, miRNAs are also capable of inducing immunotoxicity. The systemic delivery of miRNA activates the innate immune system, resulting in abrupt toxicities and significant unwanted side effects. Systemic administration of miRNA duplexes could trigger the secretion of inflammatory cytokines and type I interferons (IFNs) through Toll-like receptors (TLRs).

One of the huge issues concerning miRNA therapy is the off-target side effects of miRNAs. Since they are designed to target multiple pathways via imperfect matching in the 3′ UTR, miRNAs might cause undesirable gene silencing of multiple tumor suppressor genes. The off-target gene silencing might induce potential toxicities and subsequently reduced the therapeutic effects. A combinatorial strategy could be added to the miRNA therapy to prevent undesirable off-target effects [81]. Multifunctional nanoparticles that codelivered miRNA and siRNA that could silence certain oncogenic pathways and activate tumor suppressor miRNAs were reported to avoid off-target effects [82].

## 7. Delivery of miRNA-Based Therapeutics

To improve the efficacy of miRNA delivery, there are two main strategies: local (intratumor) or systemic delivery. Local delivery is beneficial since it required lower doses of miRNA, showed reduced toxicity, and could selectively deliver miRNAs to the target tissues [83]. Nevertheless, local delivery is only useful for solid tumors, and it is not useful in hematological malignancies such as leukemia. Besides, local delivery is also not suitable for metastasizing cancer cells observed in late-stage disease as they are not exposed to the RNA drugs in circulation. Thus, systemic delivery is a preferred route of administration as it provides greater efficiency of the biodistribution of drugs to the target tissues. Significant progress has been made in establishing systemic miRNA delivery strategies. Currently, both nonviral and viral miRNA delivery systems are used where there are specific advantages and disadvantages for each of the approaches (Table 2).

### 7.1. Nonviral Vectors

The use of nonviral vectors is an effective approach to deliver miRNAs. Nonviral vector systems have low immunogenicity and less toxicity, and there are no limitations on the size of the genes being delivered. Due to these features, nonviral delivery has become more popular and is widely applied for miRNA delivery as it can enhance the cellular uptake and the pharmacological efficacy of antisense oligonucleotides *in vivo*. Nonviral delivery vectors can be classified into three main groups including lipid-based vectors, polymeric vectors, and inorganic particle-based vectors.

#### 7.1.1. Lipid-Based Vectors

The most studied vehicles for the delivery of miRNAs are lipid-based vectors. Lipid-based approaches use the lipid complexes known as liposomes as delivery carriers. There are three types of liposomes that are based on charges such as cationic, anionic, and neutral liposomes. For example, cationic lipoplexes are most generally used in nonviral delivery systems as they have unique characteristics such as high affinity with the cell membrane, nonimmunogenic, nonpathogenic, and easier to produce. Systemic delivery of miR-29b employing cationic lipoplex into non-small cell lung cancer (NSCLC) was shown to increase the expression of miR-29b by fivefold and reduced the tumor growth rate by approximately 60% [84]. Piao et al. demonstrated the delivery of pre-miR-107 in a preclinical model of head and neck squamous cell carcinoma using cationic lipid nanoparticles, which showed 45.2% reduction of tumor growth as compared to pre-miR treatment controls [85].

Lipofectamine®, TransIT® 2020, and Oligofectamine™ are several examples of commercially available cationic liposomes capable of transporting nucleic acids into the cells [86, 87]. However, the most vital disadvantage of the liposome delivery system is the stability of the nanoparticles in sera due to nonspecific binding to serum proteins. To enhance circulatory half-life, conjugation of the lipids with hydrophilic polymers such as polyethylene glycol (PEG) could highly increase their stability, resulting in a long half-life of up to 72 hours in sera [88].

Liposomes provide the opportunity to combine miRNA delivery with various chemotherapeutic drugs that could lead to a synergistic and improved therapeutic effect. For the improvement of cisplatin therapy and drug resistance impairment, cisplatin-coated liposomes loaded with miR-375 in hepatocellular carcinoma (HCC) were utilized. The nanoparticles were developed by combining two reverse microemulsions consisting of KCl solution and a soluble *cis*-diaminedihydroplatinum (II) coated with a cationic lipid layer, with the integration of miR-375 into the lipid-coated cisplatin. *In vitro* analysis of the study revealed that this type of codelivery showed an efficient escape of miR-375 as well as induced apoptosis rate and cell cycle arrest in HCC cells [89].

#### 7.1.2. Polymer-Based Vectors

Polymeric vectors acquired an outstanding position among the nonviral vectors due to their positive properties such as low toxicity and immunogenicity and high composition variability and could be manipulated to improve cellular uptake, tissue specificity, and stability. Examples of polymeric vectors are polyethylenimine (PEI), poly lactic-co-glycolic acid (PLGA), and polyamidoamine (PAMAM). PEI is the most extensively used cationic polymer due to its high transfection efficiency. High-molecular-weight PEIs offer high transfection efficiency with high toxicity, while low-molecular-weight PEIs are more biocompatible with less efficiency. Ibrahim et al. used low-molecular-weight PEIs to deliver miR-33a mimics as well as miR-145 into colon cancer xenograft mice, and the polymeric nanoparticles with the miRNAs led to increased cell death and reduced tumor growth [90]. In addition, delivery of miR-145 encapsulated in short polyurethane and a branched polyethylenimine (PU-PEI) in combination with cisplatin reduced tumor growth and metastasis in a lung cancer mouse xenograft model [91].

PLGA is a well-known Food and Drug Administration (FDA) approved nontoxic, biocompatible, and biodegradable polymer used for drug delivery as well as for delivery of anti-miRNAs [92]. The PLGA-based delivery system was able to sustain release of drugs and support the physical adsorption of multiple targeting ligands on their surfaces. Li et al. demonstrated that treatment with PLGA-based nanoparticle/miR-221 inhibitor complexes suppressed cell growth, colony formation ability, migration, and invasion of HCC [93]. Furthermore, the PLGA polymer could also be modified. Surface modification of PLGA with PEG significantly prolonged the retention and circulation time of the particles *in vivo* [94, 95]. For example, particle surface modifications had successfully codelivered anti-miR-10b and anti-miR-21, leading to a reduction of tumor growth in mouse breast cancer xenograft [96]. One of the advantages of using PLGA in the delivery system is the high loading capacity of PLGA particles. The delivery of copolymers containing PLGA and PEI was set out by taking advantage of the effective high loading capacity of PLGA and efficient cellular uptake of PEI. Wang et al. showed that hyaluronic acid-coated PEI-PLGA nanoparticles codelivered with doxorubicin chemotherapeutic drug and miR-542-3p induced good drug uptake and caused cytotoxicity in triple-negative breast cancer cells [97].

PAMAM is a positively charged polymer with a large number of surface amino groups. Unlike the undegradable PEI, PAMAM is a biodegradable and biocompatible polymer that displays relatively low toxicity. Ren et al. demonstrated that the delivery of anti-miR-21 to human glioblastoma cells using PAMAM as a carrier could significantly induce apoptosis and decreased cell growth [98]. In addition, Conde et al. demonstrated that conjugation of RNA triple helices to PAMAM G5 dendrimer comprising miR-205 and anti-miR-221 resulted in 90% tumor size reduction and increased survival [99].

#### 7.1.3. Inorganic Material-Based Delivery

Besides lipids and polymers, inorganic nanoparticles are utilized in many studies for miRNA delivery. Examples of inorganic vectors for miRNA delivery include gold nanoparticles (AuNPs), carbon nanotubes, and iron oxide-based nanoparticles. The advantage of inorganic carriers is that they are nontoxic and nonimmunogenic, have high stability *in vivo*, and are relatively easy to manufacture. However, the common issue with inorganic nanoparticles is the interaction between the carriers and nucleic acids. Inorganic carriers have long-term colloidal stability in aqueous solutions in the absence of surfactants and nonspecific binding affinity to numerous functional groups present in biological systems [100].

AuNPs were used to effectively deliver miRNAs to breast and prostate cancer cells *in vitro*. A study by Ekin et al. designed a high-affinity gold nanoparticle-based nanocarrier modified with thiolated RNAs, and miR-145 was hybridized to the RNAs attached to the AuNPs. The AuNP-RNA-miRNA carrier complex was successfully delivered and upregulated the expression of miR-145 in both breast and prostate cancer cells [101].

Carbonate apatite presents an attractive characteristic among inorganic nanoparticles due to its biodegradability, heterogeneous charge distributions, and nanoscale particles that are easily generated [102]. Hossain et al. demonstrated efficient transfection of tumor suppressor miRNAs into colon cancer cells using the carbonate apatite-based delivery system [103]. Hiraki et al. reported that the systemic administration of carbonate apatite formulated miR-4689 dramatically inhibited tumor growth *in vivo* by directly targeting KRAS as well as AKT [104]. On the other hand, Inoue et al. also showed that in mouse xenograft models, systemic administration of miR-29b-1-5p using carbonate apatite as a delivery vehicle significantly inhibited tumor growth and proliferation of KRAS mutant colon cancer cells without any particular toxicity [105].

### 7.2. Viral Vectors

Viral-vector based systems such as retroviruses, lentiviruses, and adenoviruses or adeno-associated viruses (AVV) were modified in some specific genomic regions so that they were unable to replicate, and their safety could be increased. The advantages of viral-vector delivery systems are their high transfection efficiencies and high levels of constant expression of miRNAs or antagomirs. For example, the lentiviral miR-34a expression system was shown to significantly induce the expression of miR-34a and enhance apoptosis in multiple myeloma cells. The lentiviral vector-transduced multiple myeloma xenografts with constitutive miR-34a expression demonstrated significant growth inhibition of severe combined immunodeficient (SCID) mice [106]. Besides, the lentiviral vector also has been used to deliver miR-34a to prostate cancer cells, and data obtained showed that it inhibited tumor cell metastasis and extended animal survival [32].

The systemic delivery of miR-26a carried by the AAV and delivered into human HCC was able to cause cell cycle arrest, apoptosis of the cancer cells, and tumor growth inhibition. Moreover, the systemic administration of AAV-miR-26 showed undetectable toxicity [78]. Therefore, miRNA restoration therapy using AAVs is a safe and effective strategy for cancer therapy. Recent studies showed exosomes which are nanosized lipid vesicles that were released by virus-infected cells, and they were able to encapsulate and deliver RNA therapeutics into target cells. Pegtel et al. demonstrated that virus-infected cells packaged the virus-encoded miRNAs to exosomes, and the miRNA cargoes were later delivered into noninfected target cells [107, 108]. This indicated that exosomes could be exploited for therapeutic miRNA delivery. For example, the exosomes derived from viruses that targeted liver cells were designed to deliver therapeutic miRNAs to treat liver cancers [109].

## 8. Nanotherapy Targeting the Tumor Microenvironment

Tumor microenvironment plays an important role in controlling the distribution and biological effects of nano-chemotherapeutics. The complexity of the tumor microenvironment lowers the delivery of effective concentrations of conventional drugs to kill cancer cells. This has led to the use of different strategies involving nanoparticulate drug delivery to attain tumor specificity as well as to enhance the therapeutic index of the chemotherapeutic drugs. It is crucial to understand the biology of the tumor to design an efficient drug treatment that can defeat drug resistance, abolish tumor progression, and hinder metastasis [110]. In general, the targeting strategies concentrate on priming the tumor microenvironment to promote greater uptake of nano-chemotherapeutics and also nanocarriers targeting the tumors through the use of appropriate approaches such as receptor expression, enzymes, or tumor microenvironment modulation.

Nano-chemotherapeutics depend on the tumor vasculature in which they are extravasated into the tumor interstitium for the uptake of the drug. Nonetheless, the localization of nano-chemotherapeutics within the tumor microenvironment could also be hampered by increased interstitial fluid pressure, impaired extracellular matrix (ECM) composition, enhanced cell division, and reduced lymphatic drainage [111]. Thus, tumor-associated vasculature is one of the important targets to obtain localization of antiangiogenic chemotherapeutics for tumor growth suppression. Rapidly growing tumor vasculature is observed to have abnormal characteristics such as being irregular in shape, fragile, having tortuous structures, and dilated [112]. These abnormalities resulted in higher permeability of the vessels that lead to variability in blood distribution. Poor blood flow and increased interstitial fluid pressure led to development of hypoxic conditions as well as acidic sites. The enhanced permeability and retention (EPR) effect of macromolecules in tumors is correlated with leaky tumor vasculature. These leaky vessels promote tumor cell invasion and metastasis. All these abnormalities of tumor vasculature could impair the delivery and obstruct the efficacy of nano-chemotherapeutics [113]. A few methods have been proposed to overcome the ineffective drug delivery in the tumor microenvironment including restoration of vascular function and decompression of tumor vessels. Vascular normalization strategy has been used to remodel the tumor vasculature and brought it closer to the “normal” state. It can be conducted with the selective dosage of antiangiogenic drugs that primarily target the vascular endothelial growth factor (VEGF) or its receptors. This strategy recovers the tumor perfusion by strengthening the vessel wall as well as the structure of the vascular network that could lower interstitial fluid pressure due to decreased fluid leakage from the vessels [114]. On the other hand, decompression of tumor vessels can be accomplished with stress alleviation strategies to target the ECM (collagen and hyaluronan). This strategy could improve perfusion of collapsed vessels, lower interstitial fluid pressure by targeting the ECM component (collagen and hyaluronan), and enhance the penetration of drugs into tumor vasculature [115, 116].

Nano-chemotherapeutics targeting tumor microenvironment could be one of the successful strategies in reducing drug resistance. Several endogenous factors including acidic pH, enzyme activity, oxidative stress, hypoxia, hyperthermia, redox potential, ATP, and high interstitial fluid pressure have been considered for the effective delivery of nano-chemotherapeutics to the tumor microenvironment [117]. In addition, specific pathophysiological factors in tumor microenvironment such as different levels of amino acids, functional proteins, DNA fragments are also taken into consideration. Preclinical studies using pegylated nanocarriers, stimuli-responsive nanocarriers, and dual functional nanocarriers have demonstrated outstanding results in suppressing tumor growth by targeting the tumor microenvironment. All of these strategies include site-specific detachment of PEG linkage, the reversal of the surface-charge, particle size reduction, and susceptibility to stimuli such as pH and temperature [118–120].

Nano-chemotherapeutics could modify the delivery of drugs by promoting perturbations in the tumor microenvironment. Nanotechnology provides a flexible approach to allow delivery of single or combination of chemotherapeutics together with multiple targeting ligands to specifically target reductive environment or overexpressed receptors and enzymes, a prevalent characteristic of the tumor microenvironment [117]. Examples of ligand-mediated nano-chemotherapeutics included peptides, antibodies, carbohydrates, and aptamer linked to nanoparticles. Nano-chemotherapeutic conjugated to the functional ligand on the surface could play a significant role in enhancing drug selectivity towards overexpressed receptors, particularly to the tumor cells at all sites [117, 121]. This strategy exerts target specificity and contributed to effective treatment with minimum adverse off-target effects.

## 9. miRNA Therapeutics in Clinical Trials

miRNAs exhibit promising therapeutic potentials, and they are in the research pipelines of several pharmaceutical firms. OncomiRs or tumor suppressor miRNAs that function as master regulators of cellular processes have been evaluated in several clinical trials conducted by companies such as Mirna Therapeutics, EnGeneIC, and miRagen Therapeutics (Table 3). In May 2013, MRX34 is the most advanced miRNA mimic to enter clinical testing (ClinicalTrials.gov Identifier NCT01829971). This drug is a liposome-formulated mimic of miR-34a that acts as a tumor suppressor. miR-34a is generally downregulated in most human cancers such as breast, colon, kidney, ovary, prostate, and skin cancers [124–126]. The restoration of miR-34a has the potential to cause cell cycle arrest, senescence, and apoptosis of cancer cells [127, 128]. In June 2016, a total of 99 patients with advanced solid tumors were enrolled in the study [129]. The trial comprised a dose-escalation study with two doses per week or five doses per day administered intravenously. Each of the patients with HCC, acral melanoma, and renal cell carcinoma (RCC) achieved partial response at the end of the trial, which was evaluated through Response Evaluation Criteria in Solid Tumor (RECIST), and 14 patients were detected with stable disease. Analysis of white blood cells showed significant repression of miR-34a target genes such as forkhead box PI (FOXP1) and BCL2. However, the trial was halted in 2016 due to five severe immune-related adverse events involving death of patients [129]. This is a setback to miRNA therapy involving MRX34 and has come as a surprise due to adverse immune outcomes. In light of this halt, it becomes even clearer that effective delivery and targeting are critical to translating miRNAs into therapeutic success.

In 2014, the miR-16 mimic was developed and underwent phase I clinical trial in patients with malignant pleural mesothelioma and NSCLC who had failed in their standard therapies (ClinicalTrials.gov Identifier NCT02369198). The miR-16 mimics were delivered intravenously using EnGeneIC delivery vehicle (EDV) packaging and were conjugated with an EGFR-targeting antibody to facilitate targeting to the tumor site [130]. Preliminary data by van Zandwijk et al. showed that the treatment had a manageable safety profile in 5 patients in response to infusion of 5 billion nanocells loaded with miR-16 [131]. This TargomiR trial is expected to continue to phase II study as the results were encouraging and did not present adverse immune response and toxic effects [131].

In March 2016, a phase I clinical trial of LNA-modified anti-miR-155 (MRG-106) was initiated by MiRagen Therapeutics (ClinicalTrials.gov Identifier NCT02580552). This trial was evaluated in patients diagnosed with cutaneous T-cell lymphoma (CTCL) of the mycosis fungoides subtype, and the results were encouraging. Preliminary data demonstrated that intratumoral injection of MRG-106 resulted in improved cutaneous lesions with almost no side effects [132]. Thus, MiRagen Therapeutics in 2018 initiated a phase II clinical trial to further evaluate the efficacy of MRG-106 against CTCL (ClinicalTrials.gov Identifier NCT03713320). This strengthens the credibility of miRNA inhibition in clinical applications and could encourage potential attempts to improve anti-miRs for cancer therapy.

## 10. Conclusions

Cancers are complex diseases involving diverse regulatory mechanisms to promote their progressions. Altered expression of miRNAs from cellular events such as chromosomal DNA mutations and defects in the biogenesis of miRNAs will lead to cancers. The development of miRNAs as therapeutics is promising to be further explored for cancer treatment. miRNA inhibition and restoration therapies might be used as effective strategies for targeting and suppressing multiple oncogenic pathways. Although there are phase I and II trials to investigate miRNA-targeting drugs, to date, there is no miRNA drugs that have entered into clinical phase 3 trial. Thus, it is crucial to define the distinct expression of miRNAs in different types of cancers to further design treatment strategies and prevent off-target effects of miRNA drugs. The development of new miRNA delivery systems to specific cell types, tissues, and organs by establishing specific carriers will further improve the efficiency and specificity of miRNA therapies for cancers. Targeting miRNAs to specific cell types using antibodies, ligands, and nanoparticles have been designed, which showed enhanced specificity and reduced immunotoxicity [82]. The targeting agents such as antibodies (single-chain variable fragment, scFv; disialoganglioside, GD2), peptides, or ligands (hyaluronic acid) could improve tissue-specific delivery and biodistribution and reduce the dose delivered, further preventing delivery-associated toxicity [133, 134]. Lipid nanoparticles are usually modified with other molecules, including hyaluronic acid and PEG to enhance tumor targeting and stability [135]. Inorganic-based nanoparticles are modified by conjugation with antibodies or encapsulated in natural vesicles to produce nanoparticles with clinically relevant properties, including tumor-targeting ability [136, 137]. Since most of the inorganic nanoparticles are not naturally tumor-specific, several methods have been used to enhance the tumor-homing capability of nanoparticles such as conjugation with antibody targeting the GD2 antigens overexpressed on the surface of solid tumor cells [136], conjugation with an antibody targeting the cancer-specific antigen epidermal growth factor receptor [138], or conjugation with hyaluronic acid that could target CD44 on colon cancer cells [139]. Besides, the use of nanocarriers to deliver miRNAs is particularly beneficial in cancer therapy where they have the possibility of passive accumulation in tumor tissues due to their leaky vasculature and lowered lymphatic function known as the EPR effect [140, 141]. Despite all the challenges, miRNA-targeted therapeutics will become a potential therapeutic once a suitable miRNA delivery system with enhanced efficacy and specificity can progress from phase I/II to phase III. As cancer development is driven by multiple cellular pathways, common therapies used such as chemotherapy and radiotherapy for cancer treatments are only partially effective. Hence, combinatorial approaches with miRNAs therapy should be encouraged.

## Figures and Tables

**Table 1:** miRNAs as oncomiRs or tumor suppressors in cancers.

miRNAs	OncomiRs/tumor suppressor miRNAs	Cancer types	Function	References
miR-21	OncomiR	Lymphoma, breast, lung, colon, glioblastoma, pancreatic, ovarian, prostate, and gastric cancers	Promoted cell proliferation, metastasis, and invasion	[13–17]

miR-17-92 cluster	OncomiR	Lymphomas, lung cancer, colon cancer, and gastric cancer	Promoted angiogenesis and tumor growth	[18, 19]

miR-155	OncomiR	Lymphoma, lung, breast, and ovarian cancers	Promoted angiogenesis and tumor growth	[20–22]

miR-10b	OncomiR	Breast cancer, glioblastoma, and esophageal cancer	Promoted invasion and metastasis	[23–25]

miR-221/222	OncomiR	Lung cancer, hepatocellular carcinoma, breast cancer, gastric cancer, and glioblastoma	Promoted cell migration and proliferation	[26, 27]

Let-7	Tumor suppressor miRNA	Colon, breast, and lung cancers	Inhibited cell proliferation and regulated the cell cycle	[28, 29]

miR-34a	Tumor suppressor miRNA	Breast cancer, colon cancer, pancreatic cancer, and melanoma	Inhibited proliferation and invasion	[30–33]

miR-15a/16	Tumor suppressor miRNA	Leukemia and colorectal cancer	Promoted tumor growth	[34, 35]

miR-29	Tumor suppressor miRNA	Cervical cancer, breast cancer, and acute myeloid leukemia	Proliferation and metastasis	[36, 37]

**Table 2:** miRNA delivery strategies.

Method of delivery	Advantages	Disadvantages
*Nonviral vectors*	Low immunogenicity Low cost and easy to use	Less efficiency
(1) Lipid-based vectors	Protect RNA molecules within the vesicles to form stable nucleic acid lipid particles	Poor *in vivo* stability
(i) Liposomes		
(a) Cationic		
(b) Anionic		
(c) Neutral		
(2) Polymeric vectors	High structural and composition variability Low toxicity and easy to use Easily manipulated to increase stability, tissue specificity, and cellular uptake	Poorly biodegradable and toxic (PEI) Accumulate in the liver (PAMAM)
(i) Polyethylenimine (PEI),		
(ii) Poly lactic-co-glycolic acid (PLGA),		
(iii) Poly amidoamine (PAMAM)		
(3) Inorganic nanoparticles, e.g., carbon nanotubes (CNT), metallic nanoparticles, and nanorods based on iron oxides (IOs)	Low cytotoxicity Nonimmunogenic High stability *in vivo* Easily manufactured	Long-term colloidal stability in aqueous solutions in the absence of surfactants Nonspecific binding affinity to various functional groups

*Viral vectors*		
Adenovirus, lentivirus, and retrovirus	High transfection efficiency Stable expression	High immunogenicity High toxicity Complicated large-scale production Relatively expensive

**Table 3:** Clinical trials using miRNA therapy in human cancers.

Drug (company)	Therapeutic agent	Cancer type	Delivery system	Trial status	Clinical trials gov. Identifier
MRX34 (Mirna Therapeutics)	miR-34 mimic	NSCLC, RCC, primary liver cancer lymphoma, melanoma, multiple myeloma, and renal cell carcinoma	LNPs (Smarticles)	Phase I terminated due to immune-related toxicities and deaths	NCT01829971
MesomiR-1 (TargomiRs) (EnGeneIC)	miR-16 mimic	Malignant pleural mesothelioma and NSCLC	EnGeneIC delivered in an EDV nanocell with EGFR antibody surface conjugation	Phase I completed Expected to enter phase II	NCT02369198
MRG-106 (miRagen Therapeutics)	Anti-miR-155	Cutaneous T cell lymphoma, mycosis fungoides, chronic lymphocytic leukemia, and adult T-cell leukemia	LNA-modified antisense inhibitor	Phase I (active, not recruiting) Phase II (active, not recruiting)	NCT02580552 NCT03713320

Adapted from Balacescu et al. [122] and Rupaimoole and Slack [123]. Abbreviation: LNPs, lipid nanoparticles; NSCLC, non-small cell lung cancer; RCC, renal cell carcinoma; EDV, EnGeneIC delivery vehicle; EGFR, epidermal growth factor receptor; and LNA, locked nucleic acid.
